# "The Hospital" Nursing Mirror

**Published:** 1901-06-29

**Authors:** 


"The Hospital" Nursing Mirror.

Being the Nursing Section of "The Hospital."

[Contributions for this Section of "The Hospital" should be addressed to the Editor, "The Hospital" Nursing Mirror, 28 & 29 Southampton Street Strand, London, W.C.]

### Notes on News from the Nursing World.

### RECEPTION BY THE QUEEN OF JUBILEE NURSES.

The Queen has consented to receive the Jubilee Nurses on Wednesday next, and to give the badges to the probationers who qualified to become nurses in December last. Of these there are 193, being respectively 139 from England and Wales, 36 from Scotland, and 18 from Ireland. Her Majesty will also distribute certificates of extra proficiency and "leaving badges" to about 64 other nurses. But, in addition to the 257 nurses thus invited, the Queen, with her customary anxiety to afford gratification to as many persons as possible, has been graciously pleased to invite as many more "Queen's" Nurses all over the kingdom as can conveniently attend to participate in a sort of informal garden party in the grounds of Marlborough House. Owing to the mourning there cannot, of course, be anything like a public function, and there will be no other guests except the Council of the Queen's Jubilee Institute and the nurses themselves. The London Scottish Drill Hall has been lent to the Council for the purpose of mustering the nurses, and they will march, about 600 strong, from the Hall to Marlborough House. Princess Louise, Duchess of Argyll, as President of the Scottish Council, will be present with the Scottish nurses; and probably Lady Cadogan, as President of the Irish Council, with the Irish nurses.

### ARMY SISTERS FOR SOUTH AFRICA.

The Secretary of State for War informs us that 30 nursing sisters embarked for South Africa on the s.s. *Assaye*, namely, Miss M. G. A. Warner, of the Army Nursing Service, and the following members of the Army Nursing Service Reserve:—Mrs. A. Twamley, and the Misses E. Ambrose, L. E. V. Asman, M. L. T. Babb, M. J. Bell, A. McJ. Bower, F. A. Davis, F. de Blaquiere, L. M. Fletcher, M. H. W. Griffiths, M. M. Hanson, A. Jarman, G. E. Larner, E. C. Lloyd, M. Lynch, A. B. McDonald, M. Neville, A. O'Flaherty, M. E. Owen, M. E. Powell, A. M. Poulter, N. W. Pughe, E. J. M. Robbins, G. H. C. Stratton, A. L. Storie, M. R. Thomson, A. Thornton, G. Trout, and K. Ward. Several of these sisters have already served in South Africa, while others are going out for the first time. That it should be considered necessary to send out such a substantial number of nurses at the present time is rather ominous, though there is no doubt that they are required.

### THE WAR OFFICE AND COLONIAL NURSES.

Three weeks ago we gave prominence to a complaint from a member of the Royal British Nurses' Association to the effect that colonial nurses had been very badly treated by the War Office during the South African campaign. In a letter which we publish to-day a nursing sister testifies from personal experience that both by the Army Medical Department in Capetown and also by the War Office colonial nurses have been treated with every consideration. She avers that their pay and allowances are practically the same as those of the reserve sisters sent out from home, and that they have the advantage, if they choose, of resigning at a day's notice and having their expenses defrayed to Capetown. We note a further statement of our correspondent with surprise. She says that one of the colonial nurses attached to the hospital in which she was engaged "had barely received a year's training in a children's hospital," and that others employed by the Army Medical Department at Capetown "had only had three months' training." If this is so, it is the turn of fully-trained nurses at home who could not gain admission to the Army Nursing Service Reserve to complain, because, as it was alleged, their services were not required.

### NURSES WANTED IN CAPE COLONY.

A fortnight ago we mentioned that nurses were in great demand in some parts of Cape Colony, and, as we expected, a number of letters have been sent to us by readers who are anxious to obtain fresh particulars. Some of our correspondents ask for details which it is obviously impossible to give, and others entirely misunderstand the position of affairs. One of them, for instance, asks "How much it would cost to live in Cape Colony," and another inquires, "Would passage out be paid?" The answer to the former is that, generally speaking, living in Cape Colony is considerably more expensive than it is in England, and that accordingly it is necessary to earn more money; while as to the latter, there is no question of the payment of any expenses. The situation, as we are informed by a correspondent in Queenstown, is just this—the want of more private nurses is badly felt, and there is therefore probably an opening for two or three who have had three years' general and some fever and maternity training, possess a little capital of their own, can afford to run the risk of not meeting with immediate success, and do not mind roughing it. We can give no addresses, but the Agent-General for Cape Colony may be able to supply the names of medical men. In this connection we notice that at the annual council meeting of the Women's Liberal-Unionist Association a statement was made by Mrs. Lyttelton Gell to the effect that "a matron and a whole staff of nurses," are required to undertake a hospital at Salisbury, in Mashonaland, and that a similar condition of affairs prevails at Umtata. Monthly nurses are also, she added, much needed, and she intimated that the British Women's Emigration Association would advance money for passages to South Africa in cases where it is wanted.

### THE BISHOP OF LONDON AND THE NURSES.

At the opening of the new medical wing of the Poplar Hospital for Accidents the Bishop of London, who performed the ceremony, said in the course of an eloquent and sympathetic speech that "the Poplar Hospital was more especially in favour with the population of that district, for it meant, among other things, that the loving devotion of the nurses was always at their service, while they received there a tenderness and care which could not be surpassed in any other hospital in the world." This testimony by the Bishop to the work of the nurses is the more valuable because, as Head of Oxford House, and subsequently as Bishop of Stepney, Dr. Winnington

Ingram enjoyed so many opportunities of judging for himself the kind of feeling prevailing towards the nurses among the poor and the suffering in the East End.

### NURSES' NEW QUARTERS AT POPLAR HOSPITAL.

The new wing just opened at the Poplar Hospital for Accidents provides accommodation for six additional nurses and one sister; the latter has a charming little bed-sitting-room. The nurses' apartment is a long narrow room, wainscotted with white wood; the walls are a soft, quiet grey. It is very light, and the view from the windows takes in the masts of vessels in the docks. The nurses may be congratulated on possessing one of the quaintest of sitting-rooms, and one of the most artistic. There are plenty of cosy armchairs, and the green leather-covered fender guarding the copper-fitted fireplace harmonises pleasingly with the apple-green curtains and carpet. A door leads out on to a small balcony, whence one looks down into the chimney-pots of smaller houses below. There is a shelter on the leads above, where fresh air can be obtained in plenty, as well as a wide view of the East of London and a glimpse of country in the distance. There are now thirty nurses in the hospital, with ninety beds under their care. The additions include a small kitchen attached to each ward, a great convenience for washing up after breakfasts, &c. Those nurses who were not on duty were present at the opening ceremony; but they returned to the wards immediately after it was over, "to keep the people in order," as Mr. Sydney Holland said. They had taken great pains to make the wards gay with flowers, and one had had some lovely water-lilies sent from home for the occasion. The stream of visitors was of great interest to both patients and nurses, and the only person who seemed disconcerted was a poor mite in a scarlet jacket, who clung to the rails of its crib and howled at the strange faces. Visitors tried in vain to comfort it, but its sorrows ceased when its nurse took it in charge.

### MIDWIVES AND THE MEDICAL DEFENCE UNION.

A good deal of interest has been aroused among nurses who hold the midwifery certificate of the L.O.S. by the action of the Medical Defence Union in maintaining that these are not "diplomas" qualifying the holders to act as midwives. The fact seems to be that at the present time no "qualification" or "diploma" of any kind is required by those who choose to practise midwifery, and that so far as the legality of their position is concerned those with these so-called diplomas are no better off than those without them. We understand, however, that the Royal British Nurses' Association will retain the term "midwife" in their forthcoming list of members as applied to those who hold these certificates, but that a note will draw attention to the fact that since June 1895 the London Obstetrical Society has ceased to use the word "diploma," and hence that this word must be read as "certificate" wherever it occurs.

### NUNS AS MATRONS.

It was inevitable that the decision of the trustees of the South Charitable Infirmary and County Hospital, at Cork, to appoint as matron, in place of Miss Moss, a member of a religious order, would be only the beginning of changes; and we do not, therefore, share the surprise which was manifested by Sir John Scott and others when Mr. Howard coupled with his motion for the election of Sister Mary Albeus Fogarty, as matron, a proposal that three other nuns should be appointed as assistant matrons. It was equally a foregone conclusion that the supporters of the new departure would be driven to admit that neither the new matron, nor her three assistants, would be able to discharge certain duties appertaining to the office. This is one of the drawbacks of the system of nursing by members of a religious order. But it was very properly pointed out and emphasised by the dissenting trustees at the meeting that, if the office of matron had been thrown open to competition in the ordinary way, it would have been easy to have secured the services of a fully qualified nurse who would have undertaken all its obligations. Nor was there any objection on the part of the minority to the choice of a Roman Catholic lay matron. But this would not have answered the purpose of the majority who raised the religious question. They want to place the whole of the nursing in the hands of nuns, and we do not doubt that in due time they will accomplish their object, though Protestants have hitherto been the main supporters of the hospital. It will be their fault if the decadence of the institution dates from the time of the initiation of their mischievous and reactionary policy.

### A NEW NURSES' INSTITUTE FOR ROCHDALE.

At a representative meeting in Rochdale, under the presidency of the Mayor, all suggestions for a local memorial to Queen Victoria were set aside in favour of founding a Nurses' Institute. Mr. Radcliffe, who seconded the proposition that any fund raised should be devoted to the erection of a handsome and commodious building for "Queen's" nurses, showed how their work had developed, and is still developing. Thus, while the number of visits paid by the nurses in 1900 was 9,913, the number in the first quarter of 1901 was 3,606, an increase at the rate of 60 per cent. That the patients and their friends appreciate the services of the nurses is evidenced by the fact that last year they voluntarily contributed £21 to show their gratitude. These statements, supplemented by an intimation from Mr. Radcliffe that the present building is too small to accommodate the nurses, doubtless created an impression upon the meeting, and attempts to enlist sympathy for other projects utterly failed to accomplish their purpose.

### NURSES AND THEIR CORRESPONDENCE.

There was quite a storm in a tea-cup at the last meeting of the Gloucester Board of Guardians concerning the proposed provision of a letter-box for the more prompt delivery of the nurses' letters. This trifling change was recommended by the Permanent House Visiting Committee, one member of which stated that their only desire was that "some means might be adopted by which the nurses could receive their letters directly they were delivered instead of being left in the letter-box from morning until night." Two or three members of the Board took objection to this recommendation as a reflection on the master, who denied "ever having kept a letter undelivered or the box unopened for twelve or even six hours." It was suggested that the master could not be responsible for the house unless he was personally accountable for the receipt and delivery of all the letters through a general letter-box, and this view prevailed with the majority of the Board, who rejected the committee's recommendation. The master was, however, admonished by the chairman to secure the prompt delivery of letters to the owners as soon as they were received from the postman. That some annoyance has been felt in the past is pretty evident from the general tenor of the discussiton, and Mr. Spring, who advocated a greater consideration for the nurses in the matter of their correspondence, warned the Board that "while it was easy to get any number of masters, it was exceedingly difficult to get nurses to occupy responsible posts."

### THE NURSING QUESTION AT LURGAN.

The Lurgan Board of Guardians will at their next meeting discuss the scheme of the Irish Local Government Board which has in view the conversion of the infirmary into a training school for nurses by the appointment of a resident physician, an assistant medical officer of the house, and a superintendent nurse. The Infirmary Committee have already considered the scheme and declared in favour of it. They wisely maintain that, though the cost per year will be £41 more than under the present unrecognised system, the benefit to the patients by the increased medical and nursing facilities, and the direct saving on the probable development of the probationers into assistant nurses, outweigh the temporary financial loss. It may be hoped that the Guardians will adopt this view, instead of being content with the existing state of things.

### OPENING OF A HOME AT ST. HELENS.

The new nurses' home for the St. Helens district nurses, provided by the generosity of Mr. and Mrs. W. W. Pilkington, has just been opened. It is conveniently arranged for the purpose it is intended to serve, and when the funds of the Association permit of the engagement of more nurses there is ample room for their accommodation. At present there is a sister, who superintends the house, and is assisted by three fully-trained nurses. The organisation was affiliated last March with Queen Victoria's Jubilee Institute, and it is expected that this new departure will greatly increase the efficiency of the work. The Medical Association in the town has appointed three of its members to sit on the committee.

### BRENTFORD UNION INFIRMARY.

The matron's report of the fifth year of the Training School for Nurses at the Brentford Union Infirmary, Isleworth, shows that since July 1st, 1900, five staff nurses who completed their training have left to take up various appointments, four probationers have been promoted to be staff nurses, and one probationer resigned on account of health. Six probationer candidates entered the Training School, one of whom left at the end of the preliminary two months' trial. There are now in the "Home" five probationers in their second year and five in their first. All the staff nurses entered as probationers; three are in their fourth year, seven in their third, and two in their second year. A course of classes has been held, and practical demonstrations and instruction in massage have been given to the senior nurses, eight of whom are going up for the examination to be held next month by the Incorporated Society of Trained Masseuses. The usual class and ward teaching in bandaging and nursing subjects has been given to the nurses and probationers by the matron and ward sisters, and they are having a thorough medical and surgical training. The medical superintendent has given a course of lectures on physiology, as well as several clinical lectures, which have been a great help to the nurses. Instruction in anatomy has been given by Dr. McLachlan. Dr. Seymour Sharkey, of St. Thomas's Hospital, held the third final examination for nurses in their third year, June 8th and 17th. Dr. Sharkey writes: "The examination was very satisfactory. Both the written part and the *vivâ voce* part were done well, and all the nurses passed."

### SHEFFIELD NURSES' HOME AND INSTITUTION.

At the annual meeting of the Sheffield Nurses' Home and Training Institution, the committee reported that the nurses' earnings had been £1,570. It was pointed out that no training fees were paid. This is accounted for by the fact that instead of taking probationers to train, the committee now confine the staff to nurses who have been fully trained. Accordingly, though the training fees are saved, the expenditure in salaries is larger. But the use of the word "Training" as a portion of the title should be relinquished, and we should like to be assured that it is not intended to pay the salaries of the new district nurses it is proposed to appoint out of the earnings of the nurses of the Institution.

### THE NURSING QUESTION AT BEDWORTH.

It is unfortunate that the people in the little town of Bedworth should be at loggerheads on the nursing question. At a meeting of the supporters of the Bedworth District Nursing Association, which is in connection with the Jubilee Institute, the chairman expressed his regret that another association with their name had been started. It certainly is not fair for a new organisation to appropriate the designation of an old one. Moreover, there cannot possibly be room in Bedworth for two nursing associations, and for the sake of all, especially of the poor, we hope that harmony will soon take the place of discord.

### THE CATHEDRAL NURSE SOCIETY.

Mr. George Renwick, M.P., who presided at the annual meeting of the Cathedral Nurse and Loan Society for the sick poor of Newcastle-on-Tyne, and moved the adoption of the report, said he was glad to see that the prejudice against professional nurses which existed some years ago, had now disappeared. In fact, the position had been reversed, and in these days people were, he found, only too pleased to call in the assistance of the nurse. Mr. Renwick might have added, in regard to some persons, "especially when they do not have to pay for it." In Newcastle, happily, there is no indisposition to pay, for the financial statement of the Cathedral Nurse Society shows that the total receipts for last year amounted to close upon £2,000, and that the balance in hand exceeds £52. The cases attended numbered 1,769, the visits paid 16,595, and there were no less than 32 night nurses employed during the year. An admirable feature of the Association is the Convalescent Home at Hexham, where, for the weekly payment of 12s. for women and 8s. for girls under fourteen, any suitable case is admitted, even if the convalescent has not been a patient of the Cathedral nurses.

### SHORT ITEMS.

The Secretary of the International Congress of Nurses inform us that the Director-General of the Buffalo Exposition has appointed September 21st to be "Nurses' Associated Alumnæ Day."—Mr. W. W. Astor has sent a cheque for £5,000 to the Women's Memorial Fund in connection with Queen Victoria's Jubilee Nurses.—Nursing sisters Amy Bland Hill and Mabel Ethelind Brooker Smith were discharged from hospital in South Africa to duty in the week ending June 9th.—Lady Roberts is appealing for £20,000 on behalf of the Jubilee Fund of the Zenana Bible and Medical Mission, of which she is President. The scheme which the committee have in view embraces the effective maintenance of existing agencies, the extension of village work, the erection of another hospital, after proposals one and two have been given effect to, and the establishment of new dispensaries. Donations should be sent to the treasurer, Lord Kinnaird, 1 Pall Mall East, London, S.W.—Five nursing sisters have arrived from South Africa this week, viz., Sisters F. H. Camp, M. Newton, and F. A. Farrar, on board the ss. *Wakool*, all on leave; Colonial Nursing Sisters L. E. Coleman (leave), and W. Rooke (time expired), on the ss. *Harwarden Castle*.

### Notes of Surgical Lectures to Nurses.

Delivered at the London Temperance Hospital by Dr. W. J. COLLINS, M.S., B.Sc., D.P.H.Lond., Fellow of London University and of the Royal College of Surgeons. Surgeon to the London Temperance and the Royal Eye Hospitals.

### ABSORPTION AND EXCRETION.

ABSORPTION, or the entrance of the products of digestion into the blood, is, in the case of peptone, sugar, water, and salts, effected directly by the bloodvessels, and in the case of emulsified fat by lymphatics. The system of lymphatics resembles the circulatory system *minus* the arteries and heart, *i.e.* it consists of capillaries and afferent trunks corresponding to veins which are collected into the thoracic duct; this duct, commencing on the second lumbar vertebra, passes up the thorax behind the aorta and entering the neck pours its lymph into the left innominate vein. The lymph-capillaries form a fine network amid the cells and tissues which compose the body, terminating in open mouths or in blind extremities—in the small intestine they are known as lacteals. Lymphatic glands occur in various localities, intercepting the lymph-stream (as in the groin, axilla, neck, mesentery). Lymph, in passing through glands, loses fat and gains corpuscles. The spleen, the tonsils, and so-called adenoid tissue (*e.g.* post-nasal) resemble lymphatic glands in structure. Absorption of morbid poisons takes place similarly either by the bloodvessels or by lymphatics: an alkaloid or microbe injected or inoculated at one spot may thus be rapidly conveyed to distant parts. A drop of hydro-cyanic acid put in the mouth may kill in less than thirty seconds by action on the nervous system. A poisoned wound of the finger may occasion redness and swelling of the lymphatics of the arm, an abscess in the axilla, and death from blood-poisoning. A poison is defined as "any substance or noxious thing which, when applied to the body, without acting mechanically, but by its own inherent qualities, can destroy life" (corrosive, irritant, narcotic). Septic poisoning (pyæmia, septicæmia, sapræmia) is due to absorption of putrid matter, bacteria, or their products by the bloodvessels or lymphatics. Various forms of microscopic fungi have been associated with various specific diseases (*e.g.* anthrax, tubercle, actinomycosis). The poisons produced by bacteria are called *toxins*, and the antidotal properties held to reside in the blood of the immune are ascribed to *anti-toxins*.

The treatment of poisoning consists in checking further absorption, facilitating evacuation, and neutralising effects. To achieve the first, emetics (sulphate of zinc, or ipecacuanha, or injection of apomorphia) or stomach pump, which may be improvised out of a funnel and india-rubber tube. The antidotes for the commoner poisons are:—

| POISON. | ANTIDOTE. |
|---|---|
| Acids ... ... | ... Alkalies, such as soda or lime. |
| Alkalies ... ... | ... Acids, such as vinegar. |
| Antimony (tartar emetic) | ... Tannin, tea, or coffee. |
| Arsenic ... ... | ... Dialysed iron. |
| Belladonna (atropine) | ... Artificial respiration, hot coffee. |
| Carbolic acid ... | ... Saccharated lime water. |
| Chloral ... ... | ... Ammonia, strychnia; rouse. |
| Chloroform ... ... | ... Artificial respiration; invert, battery. |
| Corrosive sublimate (Hydrarg. perchlor.) | ... White of egg, gruel. |
| Lead ... ... ... | ... (Acute) sulphuric acid or mag. sulph.; (chronic) pot. iod. |
| Nux vomica (strychnine) | ... Bromide of potassium, chloral. |
| Opium (morphia) ... | ... Rouse, hot coffee, ammonia. |

Many bacteria are believed to produce their deleterious influence on the body by virtue of chemical poisons (alkaloids, ptomaines, toxins) they produce. The object of cleanly or aseptic surgery is to make such contamination of wounds impossible, since wounds under certain conditions may be favourable to absorption of blood-poisons. Heat (boiling), light, and chemical agencies are employed to render clean and "sterile" the instruments, sutures, dressings, skin of patient, hands of operators, and environment generally. Healthy blood contains no organisms, and, indeed, exerts a destructive influence upon them. The soil as well as the seed must be studied, and, where practicable, the general health of the patient attended to prior to operation. Moreover, different poisons—animal, vegetable, and mineral—act differently on different tissues, exerting what is called a selective action (strychnine on spinal cord; tuberculin on lupus), and there is ground for thinking there is a close relationship between chemical constitution and physiological action. Poisons are eliminated from the body through the lungs, skin, bowels, and kidneys—the routes whereby the ordinary removal of effete material from the body is accomplished.

An excretion differs from a secretion inasmuch as the latter subserves some further useful function in the animal economy. Saliva is secreted, urine is excreted; bile is both a secretion and an excretion. It has been said that every organ of the body in performing its own special function is excretory as regards all the rest.

The following table serves to summarise the income and expenditure of the body:—

| INCOME. | | | OUTGO. | | REPAIR TISSUE. |
|---|---|---|---|---|---|
| Food .. | (Digestion and absorption) | B | | | |
| | | | | | ¾ heat |
| | | L | (Assimilation and metabolism) | ENERGY | |
| | | O | | | |
| Oxygen .. | (Respiration) | | | | ¼ Muscular action (exteral and internal) |
| | | O | | | |
| | | D | | | |
| | | | | | [Excreta] |

It is estimated that three-fourths of the outgoings of the energy of the body, derived from food and oxygen assimilated by digestion and respiration, take the form of heat. The normal temperature is 98·4° Fahr., and varies with time of day, climate, season, age, sex, &c. Wherever combustion is going on (in the tissues, not in the blood) heat is being produced, notably in muscles, brain, and secreting glands. Heat is lost from the skin by conduction, radiation, and evaporation of sweat secreted by the tubular sudoriparous glands, from the lungs by warming the expired air, and by warming the excreta. By the nervous system the loss and possibly the production of heat is regulated so that the temperature of the body is kept within normal limits, largely irrespective of the temperature of its environment.

Just as carbonic acid, one of the products of the body's combustion, is got rid of through the lungs, so the nitrogenous residue of decomposed tissue and of excessive proteid diet is got rid of through the kidneys, by the excretion of urea (and a small amount of uric acid) dissolved along with certain salts, in water (urine).

Two and a half pints of urine are excreted daily (containing 500 grains of urea and 10 of uric acid). The specific gravity of normal urine is about 1020; in diabetes (grape sugar in urine) this is generally, often greatly, exceeded. The reaction is normally acid by virtue of the presence of acid phosphate of soda and uric acid; the latter is increased in gout. The kidneys, two in number, and weighing 5 ozs. apiece, are situated on either side of the upper lumbar spine; the left reaches to the upper, the right to the lower border of the corresponding eleventh rib. Two tubes, the ureters, 18 inches long, connect the pelves of the kidneys with the bladder, and conduct the urine as distilled, thither to await expulsion by micturition. In addition to the ureter the renal vein leaves and the renal artery enters the pelvis of the kidney; the network of microscopic branchings of these vessels compose the substance (parenchyma) of the kidney. The urinary tubules terminate in little blind pouches encapsuling a tuft of capillaries, the whole being no larger than $\frac{1}{120}$ inch in diameter, and known as Malpighian corpuscles. The peritoneum covers the kidney, more or less, sometimes so amply as to allow very free movement (floating kidney).

In inflammation of the kidneys (Bright's disease) blood and plasma may escape from the bloodvessels into the urinary tubules, causing albumin to appear in the urine (albuminuria), as shown by coagulation on boiling, the precipitate not disappearing on the addition of nitric acid ($HNO_3$). The presence of *blood* is detected by the production of a turquoise blue colour on pouring ozonic ether and tincture of guaiacum into a test-tube containing the urine. "*Casts*" of the urinary tubes composed of lymph blood or pus may be detected by the microscope in urinary sediment in cases of inflammation of the kidneys. *Mucus*, often present as a flocculent cloud, may be greatly increased by catarrh of the bladder or cystitis, especially if the urine be alkaline from decomposition of urea into carbonate of ammonium. *Pus* from the pelvis of the kidney (pyelitis) or bladder commonly occurs in alkaline urine as a yellow flat-topped sediment; its presence can be verified by finding pus cells under the microscope or by the production of a clot on the addition of caustic potash. *Urates* frequently deposit on standing in acid urine as a pink, reddish, or brown precipitate, clearing up on warming. *Uric acid*, if in excess, deposits in hard red crystals forming a sediment like cayenne pepper, or in concretion forming smooth, oval, nut-brown, laminated "calculus." The nucleus is generally started in the kidney, and in travelling down the ureter may cause exquisite pain (renal colic). Arrived in the bladder, such calculus may slowly grow by accretion, requiring lithotomy or opening the bladder (either above the pubes or in the perinæum) to remove the stone. *Phosphates* are commonly deposited in alkaline urine or on boiling: in either case the precipitate, unlike that of albumin, clears up on the addition of nitric acid. Phosphates may also concrete into calculi in the bladder, forming soft, friable, earthy stones. A third variety of stone is produced by the deposit of *oxalate of lime*, a crystalline deposit from acid urine: it forms a hard, tuberculated, reddish-brown, "mulberry"-like calculus. *Bile* in urine makes it mahogany-coloured, and iridescent tints are produced upon the addition of a drop of nitric acid.

### Nursing "Jack Tar" at Greenwich.

By Our Commissioner.

### A CHAT WITH THE MATRON OF THE "DREADNOUGHT" HOSPITAL.

THE conditions of nursing at the Seamen's Hospital vary considerably from those which obtain at the general hospitals. There is no nursing home, as understood in the present day, but the "nurses' quarters" form a separate portion of the building. On the occasion of my visit the other day, when the bright sunshine penetrated into every nook and corner, Miss Hall, the matron, conducted me over these quarters, in respect to which it must be said that the very best use has been made of the available accommodation. Though it is impossible to provide each nurse with a bedroom, the apartments, which are divided into two by a curtain, are spacious and airy; there are admirable sitting and dining-rooms, and an ample supply of bath-rooms. Unless or until a new hospital is erected, the comfort and convenience of the nursing staff could not be more fully considered than it is under existing circumstances. When I had been on the flat roof of the building, and had seen how some of the patients suffering from phthisis are undergoing the open-air treatment, as well as inspected the wards in one of the corridors, I asked the matron to tell me how the alteration in the system of training has worked, and whether the change from the three years in the Seamen's Hospital to two and a half, with six months' training at the Women's Hospital, Soho, has answered expectations.

"This system has worked very satisfactorily," was the reply. "We used to find that the nurses could not get appointments in women's hospitals, our patients all being men. But since the change which was made nearly three years ago we have not only had a greater number of suitable candidates for probationers, but at the end of their training they have been able to get better appointments."

### THE SPECIAL EXPERIENCE.

"I notice that one of your nurses has just been appointed matron at Bournemouth, and another night sister in London."

"Yes, they have no difficulty in securing posts when their time is up, and when our sisters leave it is usually in order to become matrons elsewhere."

"Do you have many cases of beri-beri?"

"Sometimes, when Chinese and other ships reach the Thames. Another point about the nursing at this hospital is that, there being no medical school attached to it, the nurses have to do the dressings."

"What is the number of sisters now?"

"In addition to the home sister, and the night sister, there are five ward sisters. There are also two orderlies who have special wards."

### THE CORRIDOR SYSTEM.

"How many patients are there to each nurse?"

"Just over five. The work is rather harder than in some other hospitals, and the sisters need to be excellent nurses. They have always had from four, five, to ten years' experience when they come to us. Many have been sisters at other hospitals, and it is, of course, indispensable that they should possess a three-years' certificate. The patients vary from 41 to 53 in each ward, and we should have a great deal of difficulty in managing them unless we had a suitable woman in charge of the corridor."

"Each sister is responsible for a corridor of wards?"

"Yes; and if she can manage one of our corridors, she is not likely to fail in managing a hospital."

"Is there any difficulty about the language question?"

"Scarcely any. Some of the sailors know six or seven languages, and interpret to each other, and some of the nurses who have lived abroad are acquainted with several. Now and again we have to employ an interpreter. But sailors are a most contented set of men, and it is most easy to satisfy their wants. The Swedes and Norwegians are particularly delightful."

"Are there any foreign nurses on the staff?"

"Not at the present moment, but we have had German, Swedish, and Danish nurses."

"When did you start your open-air ward on the roof?"

"In November. It accommodates twelve patients, suffering from tuberculosis, who go up there at sunrise and come down at sunset. The nursing staff consists of a sister and three nurses. The physician is very pleased with the result of the experiment."

### The Great Point.

"You have between thirty and forty probationers?"

"Yes. They come for two months on trial. The two months are not included in the three years' training. There are plenty of suitable candidates and a large number who are not at all suitable. In the first year the instruction is in practical work, and in the second the probationers have to pass an examination in physiology, anatomy, and nursing."

"Do they pass as a rule?"

"Generally. But they do not pass whether they do well or not. They must have a certain number of marks. We do not underrate the importance of theoretical training, but the great point is to get a good practical nurse."

"When they go to the Soho Hospital do they receive any remuneration?"

"No; we supply the uniform and the Soho Hospital pays their aundry expenses, but nothing more. They are occasionally asked at the end of the term to take staff work at Soho and some have done so; but we seldom keep our nurses after the period of training has expired."

"Do they ever come back to the Seamen's Hospital?"

"Sometimes to take holiday work. During the last eighteen months of training they are given work as staff nurses."

### Hours and Holidays.

"Are the hours on duty exceptionally long?"

"The probationers come on duty at 7 in the morning and go off at 9 in the evening. But they have two hours off every day during that period; once a week they are off from 2 to 6.30, and once a month from 7 one night until 10 the next. In the first six months they have a fortnight's holiday; in the second year three weeks, and in the third year a month."

"And the sisters?"

"They come on duty at 8 and go off at 10. But they have from 5 to 7 free, and they are off duty half a day once a week, and from Saturday at 2 P.M. to Monday at 10 A.M. once a month. They have a month's holiday."

"How many nurses are on duty all night?"

"One sister and nine nurses. The night sister comes on at 10 and is off at 8. She has two nights off each month, month's holiday every six months."

"Two months in the year?"

"Yes. The strain of night duty is so great that the holidays are needed. Because of that I hardly ever encourage a sister to take night duty more than a year. The day sisters stay on for several years."

### Uniforms and Pensions.

"I suppose cycling is a principal recreation."

"Most of the nurses have a bicycle, they can get into the country in twenty minutes. Then there are the river and the park; and it is quite easy for nurses to go to town and back to a matinee in their 4½ hours off duty. Outdoor uniform is optional."

"And the work is very interesting?"

"Most interesting I consider. The patients are charmin in their breadth of view respecting other nationalities. All the sailors who are well enough, whatever their nationality—Indian, Chinese, or Japanese—attend service on Sunday morning in the chapel if they wish."

"Is there any pension scheme in connection with the hospital?"

"Yes, and several of the sisters are members of the Royal National Pension Fund."

"Have many of the nurses left the seamen at Greenwich to tend the soldiers in South Africa?"

"Several have gone who were trained here."

### High Altitudes for Invalids.

By an English Nurse.

Colorado Springs, Colorado, U.S.A., as a health resort, does not seem to be so well known in England as it deserves to be, and a short account may be of interest, more especially as regards the effects of the climate on invalids. It is to-day the most attractive city of the West, beautifully situated at the foot of Pike's Peak and containing about 30,000 inhabitants. A great many of these originally came to seek health, although the object of some was the search for gold. The town is at an altitude of 6,000 feet, and for the cold-air treatment of consumption is almost unrivalled. I took a lady patient there in November from Boston. She was previously examined by some of the first lung specialists of that city, their verdict being that she had consumption in its first stage and that if she remained in Boston she could not live until March. The sputum was full of bacilli.

Upon hearing this we started almost immediately for Colorado Springs, and arrived with very little fatigue after three days' journey of the most comfortable and luxurious travelling for which America is famous. We found plenty of accommodation for invalids, both in roomy boarding houses and in small furnished houses. After looking round we concluded that it was better to take one of the latter, as contact with other invalids in a worse condition was likely to have a depressing effect. The house has a verandah in front, round which we fastened canvas awnings. This made a nice sleeping place for my patient and she began the open-air treatment at once, and continued sleeping out all winter with the exception of a few nights which were too windy. My patient never took cold, although some nights were extremely severe; for instance, on one or two the thermometer registered 16° F. below zero. Of course, a great deal of covering is required, a thick woollen gown being worn, also a hood and a flannel covering for the nose, stockings for the feet, and several hot-water bags.

The patient rapidly improved. She gained considerable weight, all the serious symptoms disappeared, several examinations of sputum made here betrayed no bacilli. This is only one case out of hundreds. Some patients are brought when almost too late for even Colorado air to effect a cure, but they are relieved and often live for years, when they could not live for more than a few months in another climate.

The weather is very changeable; summer heat, frost, snow, and cold following each other rapidly. But there is absolutely no dampness in the air, and the cold is not felt as in a more humid atmosphere. Snow disappears very quickly, leaving the ground quite dry, and there were not many days which we could not spend entirely out of doors.

### Wants and Workers.

Will anyone who has a second-hand spinal carriage either give or sell it cheaply to a man suffering from injury to the spine? He is only 26 years of age, and will never sit up or walk again. Any further particulars given by Nurse, Seghill, Northumberland.

### To Nurses.

We invite contributions from any of our readers, and shall be glad to pay for "Notes on News from the Nursing World," or for articles describing nursing experiences, or dealing with any nursing question from an original point of view. The minimum payment for contributions is 5s., but we welcome interesting contributions of a column, or a page, in length. It may be added that notices of entertainments, presentations, and deaths are not paid for, but, of course, we are always glad to receive them. All rejected manuscripts are returned in due course, and all payments for manuscripts used are made as early as possible after the beginning of each quarter.

### Some Hints on Nursing Malaria.

LECTURE BY DR. ROBERT HOWARD AT THE LIVINGSTONE EXHIBITION.

WEDNESDAY last was announced as the "Special afternoon for Nurses" at the Livingstone Exhibition, and a considerable number assembled in the lecture room to hear Dr. Howard on the nursing of malaria. The following is a summary of the lecture:—Those of you who go abroad to tropical countries will find that many of the cases you have to nurse will be some form of malaria. Many people have an idea that malaria has to be nursed in a different way to other diseases, but in reality it has to be nursed in the ordinary way.

### THE DIFFERENT KINDS.

Now a word about the different kinds of malaria. In temperate climates the usual forms are tertian and quartan ague. Both of these are characterised by short attacks lasting from four to six hours, with intervals of comparative good health. Each attack begins with shivering, then there is a hot stage with high fever and throbbing headache, and then a stage of sweating, during which the temperature rapidly falls, leaving the patient fairly well until the next attack. If you expect to find malaria cases all alike you will be very much mistaken. In malignant malaria, which is a common variety in tropical Africa, the first attack lasts very much longer; it may be thirty-six hours. Its onset, too, is generally much more gradual. In the morning the patient may be about, and even at work; by the middle of the day he wants to lie down, and by the evening the temperature is 102 or 103. Only occasionally is the onset accompanied by shivering and cold, as in tertian and quartan malaria. Should this occur, however, the nurse can do a great deal.

### DIRECTIONS TO THE NURSE.

At this stage the patient feels perfectly miserable; he has a vague undefined horror that he is going to die, a feeling which cannot be accounted for. Never leave a patient in a rigor, but direct all your attention to keeping him warm. Do not be content with one hot-water bottle. Get four or five, and put them to the feet, back, and under the arm-pits, and put on from three to five blankets. If you do it by halves, you are simply prolonging the patient's misery, whereas prompt treatment materially shortens it. Then comes the period of fever. This lasts from 24 to 36 hours. And here remember that the fever in itself is not harmful. It is simply the natural reaction of the body to the malarial poison, and therefore a moderate degree of fever does no harm. Get that clear. Of course, if the temperature goes up, say to 106 or 107, that is a special matter, and needs special treatment. But in ordinary cases avoid giving antipyrine or phenacetin by routine in order to bring down the temperature. Also avoid keeping on extra blankets in order to "make the patient perspire." You do not want to bring down the temperature by artificial means. The patient often suffers from headache; a slight degree of headache calls for no treatment, but should it become intense, relief may often be afforded by giving small doses, *e.g.*, ten grains of antipyrine or phenacetin combined with three or four grains of caffeine. Such a dose has no appreciable effect in reducing the temperature, but it generally relieves the headache.

### THE PATIENT NOT TO BE FORCED TO FEED.

Do not force the patient to eat or drink; it is a great mistake. Supposing the temperature is 103 or 104, and there is no appetite, there is no point in insisting on his taking food. Of course, if it is a long illness, and the patient is becoming exhausted, it is a different thing; but in an ordinary attack do not force him if he has a great objection to food. It will do no harm to go practically without food for a day or a day and a half. There is often intense pain in the muscles of the back; you can alleviate it sometimes by rubbing, or, if the patient cannot bear to be touched, by hot fomentations or by painting with iodine. And you can shift the pillows to make him more comfortable. For air-cushions for tropical use I very strongly recommend those made of varnished Japanese paper. I used some for two years, and they were perfectly good at the end of that time. Rubber air-cushions are not satisfactory in the tropics.

### FALLING TEMPERATURE.

Lastly, there is the stage of falling temperature. In some patients the temperature comes down with scarcely any perspiration at all, while others perspire so much that the bedclothes and garments are wet as soon as you have changed them. Should one insist on the wearing of flannel next the skin? One can lay down no absolute rule. Most people can and should wear it; others are perfectly miserable in it. If there is little perspiration, there is probably no harm in letting them wear cotton. Jæger sheets are often useful where sleeping between blankets is objected to.

### THE USE OF DRUGS.

So far I have said nothing about drugs. In most patients there is constipation, and the bowels should be kept relaxed with gentle purgatives. Sometimes there is a tendency to diarrhœa at the beginning of the attack, so that no purge is required. And then give quinine. There is no drug the use of which is more abused. It is expected to act more like a charm than a medicine, and because it is not uniformly successful in all cases it is laid aside as useless, or administered in a hopelessly casual manner. It is no good giving a dose or two one day and then leaving off; it must be given systematically and regularly. Remember that the malaria parasite has to be driven out of the system. It is not much good giving it while the temperature is still rising. My rule is to wait until the temperature is stationary or beginning to fall. And you ought to give a least 20 grains in the twenty-four hours, and continue until the attack is quite over. You will find great opposition to this, but don't be misled; treat with quinine regularly and systematically. Do not use sulphate of quinine; it is extremely insoluble. The bisulphate is more soluble, but contains a very small percentage of quinine. For ordinary use the hydrobromate is best. It is well also to have the bi-hydrochlorate in stock. This is extremely soluble, and should be given in cases when frequent vomiting makes the administration of any drug difficult. It can also be used as an enema or hypodermically. It is not liked for ordinary use because it is so soluble that patients complain that the taste of the tabloid lasts for some time after the dose is given.

### ANOTHER FORM OF MALARIA.

Generally speaking, a patient should be kept in bed for at least twenty-four hours after the temperature has fallen and remains normal or sub-normal. Apart from definite attacks of fever, there is another form of malaria often met with in the tropics. People will tell you that they have not got fever, but that they "feel as if fever were hanging about them." The malarial parasites are present in the blood, but not in sufficient numbers to cause a definite attack. The patient is usually told to "take a single ten-grain dose of quinine." Such treatment is quite ineffectual. It may kill off some of the parasites and postpone some of the symptoms; but what you really want to do is to clear the blood of parasites altogether; and for this you must give regular doses of quinine, *e.g.*, 20 grains the first day, 20 grains the second day, and the same on the third, up to the sixth. It must be a regular course, not merely occasional doses when the patient feels inclined. Hyperpyrexia is a complication which sometimes occurs, and demands treatment with cold baths. Supposing you have no bath, there are two means of improvising one. Either get a hole dug in the ground and put water in it, or get four natives to hold up the four corners of a waterproof ground sheet and fill it with water.

### A Special Complication.

So much for ordinary cases. But in Africa you meet with a special complication, viz., hæmoglobinuria, or blackwater fever. Do not call it hæmaturia. When first described it was so called, and this has misled people into endeavouring to treat it like acute Bright's disease. Also true hæmaturia is very common among the natives, whereas blackwater fever is practically unknown. To begin with, it is very like ordinary malaria. Then about the third day the patient is seized with a severe rigor, with vomiting and collapse. A few hours after he becomes yellow and passes porter-coloured urine. In order to treat this condition it is necessary to consider what has happened. For some reason, at present unknown, there has been a sudden destruction of red blood corpuscles. The contained hæmoglobin has been set free in the blood, and, being no longer of any use, has to be got rid of by the urine. The patient, who was probably anæmic before from previous attacks of malaria and residence for a year or more in the tropics, has suddenly suffered from what is practically a severe hæmorrhage. At the same time his kidneys are overtaxed with the effort of getting rid of the hæmoglobin, which is apt to get deposited in the tubules and block them up.

### The Essentials.

The three essentials are rest, fluids, and nourishment. The patient is often very restless; you must keep him quiet. Generally I think a hypodermic injection of morphia should be given, but you must be very careful on account of the condition of the kidneys; I should say give a quarter of a grain in the 24 hours. It will also help to allay the vomiting. The patient has a great desire to drink; give him all he wants. If the vomiting is very severe, feed by the rectum, and give enemata of salt and water. Keep up the liquids in order to maintain a free flow of urine and wash out the kidneys, and give all the nourishment you can. Also give stimulants—brandy first, and keep others in reserve. Give a mild purgative, followed by an enema. Then watch the pulse; it is the surest indication. And watch also the urine; the duration of the hæmoglobinuria is an indication of the amount of blood destruction that has taken place. If the pulse begins to go to pieces give more stimulants, and strychnine and digitalis hypodermically. Remember that you have to save your patients before they appear to be very ill, while the pulse is not over 120. You must not wait till they are collapsed, or they will slip through your hands.

### Reasonable Nursing.

Much can be done in this disease by reasonable nursing. Modern statistics show this, and in most cases you will get satisfactory results. With regard to drugs, I may mention that bicarbonate of soda, in doses of 10 grains to an ounce of water every hour, helps to allay the vomiting, and washes out the kidneys. Boracic acid, in similar doses, is used on the West Coast of Africa. I have not personally experienced it, but I hear that it is used with good results. Turpentine has been recommended by some, but should be avoided, as liable to irritate the kidneys and to increase the vomiting. Lastly, as to quinine. The modern view is that it should not be given. Malarial parasites are present during the first two days, but the process of blood destruction which causes the hæmoglobinuria also kills the parasites, so that quinine is no longer required.

### Everybody's Opinion.

**[Correspondence on all subjects is invited, but we cannot in any way be responsible for the opinions expressed by our correspondents. No communication can be entertained if the name and address of the correspondent is not given, as a guarantee of good faith but not necessarily for publication, or unless one side of the paper only is written on.]**

### ILL-CHOSEN NURSES.

"N. L. S." writes: I, like "L. O. S.," have also been for several years in the nursing world, but my experience has taught me that the narrow-minded sisters and matrons who would allow personalities to interfere with their judgment of a nurse's practical work are few and far between. A matron who sees a nurse at all hours, both on and off duty, must know her value better than a doctor who probably sees her for certainly not more than an hour a day. The whole tone of your correspondent's letter suggests jealousy; perhaps she hasn't a matron's testimonial, and "the grapes are sour."

### NURSES AND PRESENTS.

"Once a Nurse" writes: I wish to ask if it is against the rules of the Society of Jubilee Nurses to allow one of their workers to receive a present on retiring from a district? A friend of mine has worked in this town for three years with untiring devotion, and leaves behind her the love and esteem of all who have known her. Grateful patients desire to show their gratitude by giving her a present. The vicar and another have been told of this. Both say that it is not allowed for a Jubilee Nurse to receive a present. This, to me, seems very hard and unjust, for such tokens as these often bring joy to a nurse when she has left the great army of nurses. If a nurse marries and has little ones, it is with pride she shows her children little reminders of bygone days, and often associated with them are memories sweet to recall and beneficial to her listeners to hear related. To take from a nurse that joy seems cruel in the extreme, and if Jubilee Nurses are debarred from it I am glad that I was not one.

### A TACTLESS ADVERTISEMENT.

"A. E. R." writes: An advertisement in The Hospital, June 8, for a trained nurse in a cottage hospital, states that there are six beds, "and a servant is kept." Now where is the necessity for this statement? Is the case (of keeping a servant) exceptional? Is a nurse supposed to scrub the steps in the morning, clean the grates, &c., before seeing to the patients? Is a nurse the person from whom housework is naturally looked for? Would any hospital advertise for a house surgeon, and add "a porter is kept," thereby giving the H. S. to understand that he would not be expected to bath the male patients, clean the boots, knives, &c. When will nurses be given some standing different from general servants? When will it be recognised that Mrs. Gamp has been wiped off the slate, and that nowadays women of intelligence and education have taken her place, and that "hospital nurse" does not now mean one class of domestic servant? I wonder how many nurses felt humiliated as I did on reading that tactless advertisement.

### COLONIAL NURSES AND THE WAR OFFICE.

"Nursing Sister" writes: Seeing in a recent issue of your paper some remarks on some complaints by a Colonial nurse, on her treatment by the War Office, perhaps you will allow me to make a few statements on the other side. Colonial nurses are engaged at Capetown by the Army Medical Department there. Their pay and allowances practically are the same as those of the reserve sisters sent out from home. But they have one great advantage. They can resign at a day's notice, and have their expenses defrayed to Capetown. Moreover, as a rule they can have a free passage home for "Duty on the Voyage," and are entitled to passage back to Capetown. One of the Colonial nurses attached to our hospital previous to her engagement had had barely a year's training in a children's hospital. At our most busy time last December, she resigned and departed to Capetown, her night duty being then due. She stayed a month and coolly came back, having got herself re-engaged. The reserve sister, on the contrary, can only obtain leave with great difficulty, and on medical grounds. As far as my observations go, some Colonials were fully trained. Others were not, their training varying from full three years to three months, L.O.S., &c. As to treatment at the War Office, on arrival home I reported myself there in mufti and had a most courteous reception by the officials. Regarding Colonial sisters and the reserve, several of the former have been appointed to the latter at Capetown on producing the necessary credentials. Others, however, prefer to remain Colonial nurses since, as I have shown, they are able to resign at short notice.

### THE ANGLO-AMERICAN NURSING HOME AT ROME.

"WANDERER" writes: In reference to the Anglo-American Nursing Home at Rome, for which a concert was held in London last week, I should like to say that Miss M. E. Gibbons, of Middlesex Hospital (late matron of Tewkesbury Hospital), did yeoman's work in starting and organising the institution, and when—in January, 1901—she handed the management over to her no less able and devoted successor the committee much regretted that home calls led to the severance of the connection, and I hear that the outgoing directress carried away a specimen of Roman goldsmith's craft as a token of her committee's appreciation of her work. All who have had the ill-fortune to be sick and the good fortune then to be nursed at the Anglo-American Nursing Home, have left it full of gratitude and contentment with its management and position. As to the latter, it stands in a garden, with lovely views over the surrounding Campagna, free from the noise and bustle of the town, and yet within reach of all the advantages of a city. The committee have no easy task before them, as I understand that there is still a balance of the purchase money, amounting to £1,505, to be paid off within the next three years. Once relieved of this burden the home has every prospect of meeting its other liabilities and becoming in due course an almost self-supporting concern.

### Appointments.

ASTON UNION WORKHOUSE INFIRMARY.—Miss Annie Willis, has been appointed charge nurse. She was trained at St. Pancras Infirmary, where she was afterwards charge nurse nurse. She has since been engaged in private nursing.

BRISTOL ROYAL HOSPITAL FOR SICK CHILDREN.—Miss Constance Morgan has been appointed assistant matron, Miss Poole night superintendent, and Miss Peake sister. Miss Morgan was trained at East Dulwich Infirmary, and has since been charge sister. She has also been charge nurse at the South-Western Fever Hospital, and night superintendent at the Bristol Royal Hospital for Sick Children. Miss Poole was trained at Preston Royal Infirmary, and has since been staff nurse at the Monsall Female Hospital, sister at the Chorlton Infirmary, and charge nurse of the City Hospital, Grafton Street, Liverpool. Miss Peake was trained at the Bristol Royal Hospital for Sick Children and Bristol General Hospital.

BROOK HOSPITAL, SHOOTERS HILL.—Miss Margaret McLellan has been appointed night superintendent, and Miss Nancy Hetty Thorpe assistant matron. Miss McLellan was trained at the Royal Infirmary, Perth, and was afterwards charge nurse in the medical and surgical wards. She has since been (for four and a half years) charge nurse at the Brook Hospital. Miss Thorpe was trained at Charing Cross and Metropolitan Hospitals in connection with St. John's House, Norfolk Street, Strand. Subsequently, she was charge nurse at the North-Eastern Hospital, charge nurse for three years, and night superintendent at Brook Hospital for two years. Miss Thorpe holds the L.O.S. certificate.

EBBW VALE COTTAGE HOSPITAL.—Miss M. Bemrose has been appointed matron.

ISOLATION HOSPITAL, BARKING.—Miss A. M. Smith has been appointed matron. She was trained at the Middlesex Hospital, and has since been charge nurse at the Eastern Fever Hospital, head nurse at the Mall Infirmary, Kingston-on-Thames, district nurse (Q. V. J. I.), sister at the City of Worcester Isolation Hospital, and sister at Enfield Urban Hospital, Winchmore Hill.

SAMARITAN HOSPITAL FOR WOMEN, LIVERPOOL.—Miss Lattey has been appointed nurse matron. She was lately at the Government Hospital at Cairo, and she had charge of the gynæcological and midwifery section at the Kasr-el-Aini Hospital, Cairo.

ST. MARY'S NEW INFIRMARY, HIGHGATE HILL.—Miss Alice E. Little has been appointed matron. She was trained at St. Mary's Hospital, London, and has since been sister at Paddington Infirmary, night superintendent at St. Saviour's Infirmary, assistant matron at Kent County Asylum, Canterbury, and assistant matron at St. Olave's Infirmary, Rotherhithe, S.E.

WREXHAM INFIRMARY.—Miss R. E. Merrett has been appointed matron. She was trained at Guy's Hospital, London, and has since been assistant matron at St. Mary's Hospital, Manchester, and matron at the Falmouth Hospital and Dispensary.

### Death in Our Ranks.

NURSE SELINA NORMAN passed quietly away on June 15th, at Great Yarmouth, after a long and painful illness borne with much patience and fortitude. She was trained at Bootle, and afterwards worked for four-and-a-half years as district nurse at Longwood, near Huddersfield, which position she resigned last autumn on account of failing health. Miss Norman was much beloved and respected by all with whom she worked, as well as by a numerous circle outside, and was the recipient of several handsome gifts from her committee and others.

### TRAVEL NOTES AND QUERIES.

IS KNOKKE PICTURESQUE? (Nadar).—No, it most certainly is not, it is flat and uninteresting, but its merit consists in its splendid air, and its proximity to the interesting cities of Bruges, Middleburg, &c. . . . Full particulars in NURSING MIRROR for September 1st, 1900 See also answer to F. D. Rectory last week. All particulars of the Rhine, in articles of August 4th, 1900, and July 22nd, 1899. I think you would find more of what you seem to wish in the valley of the Moselle; two articles on this locality are about to come out, and will give you all particulars. If you cannot wait, write to me again. You would find the Moselle cheaper than the Rhine in accommodation, and in my poor opinion preferable; it is not nearly so tourist-ridden, and much more unsophisticated. Most delightful sketching is to be had. I think it is just what you want. Good accommodation from 3 marks per day. If you choose the Rhine, I think Andernach would suit you better than Königswinter.

BRITTANY IN AUGUST (Orsino).—The three articles you ask for are "An Easy Trip for Nurses" published May 6th and May 13th, 1899, and "Round Brittany in a Fortnight" October 7th, 1899. One is North Brittany and the other gives South as well. When you have read these and decided whether you prefer north or south, write to me again and say exactly how much you can spend each, and then I can help you so much better. Personally I think it spoils one's pleasure to try and do so much; I recommend you to choose one or the other but not try both. Yes, you must join the C.T.C., annual subscription, 3s. 6d., entrance fee 1s. Address Secretary, C.T.C., 139 Fleet Street, E.C. Thanks for what you say about the Roman articles, I hope to continue them in the winter. It is most encouraging when one gives pleasure.

PYRENEAN WATERING-PLACES (Bridge).—You must not arrange anything without consulting your medical man; both Eaux-Bonnes and Eaux-Chandes are frequented for the cause you mention, but there is very considerable difference in the properties of their waters, and you must not take either without advice. From the point of view simply of a summer change, personally I prefer Eaux Chandes, but they are near together. Eaux-Bonnes is situated 500 feet higher than Eaux-Chandes. The route is by Bordeaux, Pau, and Laruns, thence by carriage for half an hour. The country is most beautiful, and quite unlike Switzerland; moreover, the tripper element is totally absent.

### Echoes from the Outside World.

### AN OPEN LETTER TO A HOSPITAL NURSE.

We all have a tender spot in our hearts for little Prince Edward, and like to hear of his doings. The first birthday without his mother and father to give him greeting fell on Sunday, and the King celebrated his grandson's attainment of seven years by giving him a tiny bicycle built especially for him. Knowing that boys will be boys, and that the beauties of decoration are entirely lost upon them, his Majesty has had the machine made extremely plain, enamelled only in black, with cork handles with silver tips. The young Prince is to be taught by one of the royal footmen in the gardens of Buckingham Palace, but I conclude he will not be allowed to do much riding of a serious character, as it is not, I believe, considered advisable whilst the bones are so unformed, and therefore so easily trained into the wrong direction. Most of the evils which happen to the juvenile cyclist are due to practising not wisely, but too well.

Someone was asking the other day whether, after all, the Millenary of King Alfred, which was to have been held this year, had been definitely abandoned because lately nothing had been heard of it. Not at all. A meeting on the subject has recently taken place at the Mansion House under the presidency of the Lord Mayor, and it was then announced that the King had consented to become patron of the national commemoration, which will be held at Winchester in the second or third week in September. The statue of the famous King of a thousand years ago, is being completed by Mr. Hamo Thornycroft, R.A., but owing to its colossal size it will not be ready for unveiling before the early autumn. Two rough-hewn granite blocks will form the pedestal, one of which weighs forty, and the other thirty, tons. These are now only awaiting transit from Cornwall. A meeting of learned societies will be held at Winchester the week of the unveiling, and delegates from the Royal Societies of England and from the leading universities of Great Britain, America, and the Colonies will attend. A sum of £1,500 is still required to complete the permanent memorial and the work already undertaken, and it is hoped that it may be forthcoming before the time of the meeting so that the festivities may not be overshadowed with a cloud of debt. The people of Winchester will have cause to rejoice that they have been so closely associated with the good king, for thirty-five acres of land are being bought in his honour by the Corporation for the purpose of a public park and recreation ground, which will include a portion of the site of the once famous Hyde Abbey. Here King Alfred, his wife, and the first King Edward were buried. Excavations have been begun and already the eastern apse of the abbey has been uncovered.

It is refreshing to find that after all the thousands of pounds which have been subscribed for the sick and suffering in South Africa, the starving in India, the soldiers' and sailors' families at home, and the numerous memorials to Queen Victoria, there is still unlimited generosity simply awaiting a call when the cause needing assistance is a national one. The Lifeboat Fund has been in low water for some time, and the Duchess of Sutherland, who has come to the rescue, must be immensely pleased that her kindness has had so substantial a result. It is no small thing to hand over such a magnificent place as Stafford House to the mercy of a large crowd even for a single night, and the sum of £2 2s. admission was not sufficiently high to keep out the well-to-do general public, because, not only was there the house to see and the society to stare at, but the pastoral play in the open air to enjoy. With regard to the concert Melba alone is a name to conjure with, and 490 tickets were sold at £3 3s. each, but of course the chance of buying a table for one's guests to sup in the house of a duchess was the most sought after. £50 was the sum asked for the privilege, and the ten tables were sold so quickly that the balcony over the front entrance and immediately adjoining the banquet hall was also utilised, and the smaller tables placed there, to seat six persons, were disposed of for £25 each. Programmes with a special poem on "Lifeboat Jim" were priced at 10s. 6d. each, and so that all tastes should be catered for, an hour's music-hall entertainment was introduced during the evening. It is to be hoped that there will be a really large sum to hand over to the Fund, as numerous donations have also come in from all sides. The need of support was accidentally emphasised on the Monday before the *fête* when the papers were full of the particulars of the loss of the barque *Falkland* off the Scilly Isles, where the St. Agnes and the St. Mary lifeboats played such an important part in rescuing some of the shipwrecked crew.

To have composed music in four reigns is a unique distinction, and would almost have sufficed in itself to have made Mr. Charles Salaman famous had he never written "I arise from dreams of thee!" which is well known and loved, I suppose, by every student of good music. Mr. Salaman was eighty-seven when he died on Sunday evening, and he composed and published two songs during the present reign, though his first works were given to the public when George IV. was on the throne. He was born a few years only after Haydn's death; had met and conversed with Mozart's widow; was a personal friend of Mendelssohn, Schubert, Chopin, Meyerbeer, and Spohr; and Grisi made her first appearance in London under his management. He was but ten years of age when he was entered as a student at the Royal Academy of Music, and even till a few days before his death he played the piano with a ringing touch and a wondrous facility, and was never so happy as when he was improvising. Strangely enough, his last words were those of his famous song. Mr. Barton McGuckin, an old and intimate friend, had called to inquire how he was. The fact had been mentioned to the invalid, and he was heard to murmur, "I arise from dreams of thee!" He spoke no more.

The latest proposed development in France is distinctly amusing. Women have obtained the right to take legal degrees, and have been called to the Bar with decided success. The men, apparently angry that their wives and sisters should be able to enjoy legal modes of making money, and yet not be subjected to legal methods of losing it, have secured the assistance of a deputy who announces his intention of bringing in a Bill during the present session of Parliament making it not only possible for women to sit as jurors, but obligatory, just as in the case of men. He, however, does not apparently place implicit faith in the impartiality or the wisdom of their judgment, for he proposes that all juries shall be required to consist of six good men and true, and six women with equally unimpeachable characters. The same rules are to apply to the needful attendance, and the same fines are to be inflicted for failure to appear. Nothing seems to be said as to the foreman or the forewoman of the jury. Will the chivalry of the men lead to their unanimously selecting the best looking of the women as their leader, or if a proportion, including the other women prefer one of the gentler sex to guide them, will the other men refuse to allow such a state to exist as derogatory to their dignity? It is said that in France the women will be very glad if the proposed measure becomes law. Probably most Englishwomen would also like to exercise such a novel right until it came to a hanging case, and then—sudden illness might, I fancy, prevent their being present towards the end of the case.

The world moves on, and naturally change follows change, as it needs must do. But nothing in a small way has impressed me so much as the election of Mrs. Craigie ("John Oliver Hobbes") as a member of the Council of the Society of Authors in the place of the late Miss Yonge. Mrs. Craigie is a distinguished author whose books and plays have brought her so much repute that it was quite natural that the votes should fall to her; but those who know Miss Yonge's works—and who does not?—and remember her quiet life in her quiet home far removed from the fashionable haunts of men, cannot fail to smile at the fact that her place in the Society of Authors should be filled by a writer of brilliant *mots* and up-to-date epigrams, who is always at her best when writing of that "smart society" of which Miss Yonge had scarcely even heard.

### A Book and its Story.

### AN AMERICAN WOMAN IN IRELAND.*

IT was with genuine pleasure that our eye caught the name of "Penelope" on the attractive cover of the book before us, green as the Isle whose charms it sings, and gay as the girls and boys, whose very failings under the kindly touch of Penelope, become fascinations. On this occasion she is, as before, accompanied by her friends Francesca and Salemina, and since we last met them in Scotland Penelope has annexed a countryman of her own, and is now Mrs. Beresford. Her husband, she tells us, "Belongs to a type that is physically vigorous, intellectually full of vitality;" spiritually he is almost too good for earth. Financially his banking account is moderately good, and its balance would be larger were it not that there is never an hour when Mr. William Beresford is not signing notes and bonds for less fortunate men, giving small loans "just to help a fellow over a hard place," educating friends' children, starting them in business or securing appointments for them. The widow and the orphan have worn such an obvious path to his office and residence that no bereaved person could possibly lose his way; as a matter of fact nobody does." Mr. Beresford has been called to America to administer the estate of a college chum who, in addition to other trusts has confided his only child to his care "as a sacred charge with full power." We are not told how his own affairs were directed in his absence, for his two junior partners, who were Penelope's idea, had both failed physically, one had betaken himself to a long sea voyage, and the other to a course of brine baths, but then "it was impossible that Willie should have selected sound, robust partners . . . So accordingly he chose two delightful, estimable, frail gentlemen who needed comfortable incomes in conjunction with light duties." This absence of Mr. Beresford is the explanation of Penelope's finding herself travelling singly again. Charming Francesca, whose bright eyes and smart repartee had melted the heart of the Scotch minister, Ronald Macdonald, is now engaged to him—on probation—with the consent of her father whose memories of past flirtations, divagations, plans for a life of single blessedness, conspired to make him incredulous, but the loyal Salemina had elected to remain by Francesca's side during the time when her friend's affections were supposed to be crystalising. Salemina alone of the three was unappropriated, but with the promise of a romance not very far off.

Penelope's fears that they would not find Ireland "as Irish as they wished it to be," were dispelled the moment they set foot on her soil. They, of course, "longed for the new Ireland as fervently as any of her patriots, but wished to see the old Ireland before her peculiar and pathetic charm was lost in successive layers of Irish-American virtues in which those of thrift, punctuality, and cleanliness would be conspicuous."

"There is plenty of Old Ireland left (alas! the old patriot would say) and Dublin was as dear and dirty as when Lady Morgan first called it so, long years ago. The boat was met by a crowd of ragged gossoons, most of them barefooted, some of them stockingless, and in men's shoes, several of them with flowers in their unspeakable hats and caps." As a mode of conveyance the Irish car appealed particularly to the fair trio. The following definition of its construction is by one of Ireland's own boys. Cars be it known are of two kinds—outside and inside. "An outside car has its wheels practically inside its body, but an inside car carries its wheels outside." It has been called "Cupid's own conveyance," but this is open to question. In writing of Queen Victoria's last visit to Ireland, Penelope quotes her friend the Dean who says "Irish disaffection is after all but skin deep, it is a cutaneous malady produced by external irritants. Below the surface there is a deep spring of personal loyalty which needs only a touch like that of the prophet's wand to enable it to rush forth in healing floods. . . ." Never had the visitors heard anything like the greeting accorded to her Majesty as under miles of red, white, and blue flags she drove through the surging mass of people. Crimean soldiers, old crones in rags, gentry and peasants, all with one voice welcomed "the little lady." A woman in the crowd exclaims, "Look at her size now, sittin' in her grand carriage no bigger than me own Kitty and always in black, the darlin'."

Such a reception was one that would appeal irresistibly to American hearts. "The first cheers were faint and broken and emotion quivered on every face, and tears gleamed in a thousand eyes. It was the most touching spectacle in the world. . . . The Irish seem to me to be most loyal people; they desire nothing better than to be loyal if any persons to whom they can be loyal are presented to them."

Country life in rural Ireland was a phase that must be studied. Here is an extract. Having arrived as paying guests at Knockarney House they have their first taste of Irish housekeeping. The Irish lady presiding over her establishment, but recently dedicated to the reception of P.G.'s, is a specimen of a type not uncommon to the race whether in her own country or transported to this. Mrs. Mullarkey, "whose distinguishing characteristic after her untidiness is her suavity," has one morning failed to have breakfast at the hour arranged, and patience becoming exhausted Penelope in despair opened an attack on the regions sacred to the Mullarkey family, and the culinary department. "I opened the door leading from the hall into the back part of the establishment, but closed it hastily, having interrupted the toilets of three young children and of Mr. Mullarkey, whom I had thought dead many years. The meals were helter-skelter, the windows were supported on decrepit tennis racquets and worn-out brushes. The blinds refused to go up or down, the chairs have weak legs, the knobs of the doors were dissociated from their handles." On one occasion when bidden to the hospitable board of Lady Killbally, of Balkilly Castle, a catastrophe occurred in consequence of the faulty condition of the door-handle which was particularly exasperating. Salemina descended inside just as she was entering to finish her toilet. The room contained all that was precious to its completion. No one seemed equal to the occasion. The rod of connection having gone also various articles were produced to supply its place in vain. Curling irons, shoe hooks, lead pencils, crochet needles. The door refused to be coaxed. So in sadness Penelope and Francesca departed alone to the feast. After dinner they were surprised to find Salemina seated in the drawing-room "in unexceptionable attire." The narration of her experiences since they parted was exciting indeed. Through the wit of her maid, a country woman whom they had adopted on their travels, known to themselves as "the Derelict," Salemina's room had been boarded from outside by the means of the dining-room table, the carving-table, and a dressing-table, &c., while Peter, Mrs. Mullarkey's factotum, with the aid of Norah and Molly, held on to keep all steady. "Rooney's donkey-cart," the only available vehicle, was quickly on the spot, and Miss Salemina Peabody, after a four-mile drive, arrived, like Cinderella, before all the fun was over. "Penelope's experiences" abound in amusing incidents. They are really mirth-provoking, and should be read by all who are interested in the subject of them. Mrs. Wiggin is a keen observer, and writes with force and much individual charm.

* "Penelope's Irish Experiences." By Kate Douglas Wiggin. (Publishers: Gay and Bird, London. 6s.)

### For Reading to the Sick.

"IN YOUR PATIENCE POSSESS YE YOUR SOULS."

TAKE me from the thrall
Of passionate hopes, be Thou my All in All;
So may obedience lead me by the hand
Into Thine inner shrine and secret hall.
Thence hath Thy Voice gone forth o'er sea and land,
And all that Voice may hear—but none can understand
Save the obedient. *Isaac Williams.*

In "pastures green"? Not always; sometimes He
Who knoweth best, in kindness leadeth me
In weary ways, where heavy shadows be.

So, whether on the hill-tops high and fair
I dwell, or in the sunless valleys, where
The shadows lie, what matter? He is there.

*Henry H. Barry.*

Art thou chafing under the hand of thy God? Art thou murmuring that He should seem to look on complacently while thy desire is being thwarted, while thy will is being denied? Knowest thou not that there is a pain which gives cause for rejoicing? No conscience can feel the wound of sin but the tender conscience; no spirit can perceive its own unrest but the regenerated spirit.—*George Matheson.*

Take thy past unto the Lord again, as thou dost take thy sins, thy cares, thy sorrows, nay, thy very self, and trust Him with it. And He, Whose mighty heart, so full of love o'erflowing to His own, He Who Himself hath suffered for us, Who is "slow to anger," and "of great mercy"—Whose we are by the great triple claims of creation and redemption and adoption—He will pardon us, He will never let us go (till we wish Him to do so), He will keep us by His power, even from the black results and stalking shadows we have cast behind us as we came along life's path, and lead us safely to the bourne of rest, where sin itself shall be forgotten and cast out.—*E. A. D.*

You cannot trust God too much. You cannot be too confident that He Himself is guiding you, and that every embarrassment in your thoughts, every complication in your circumstances, is known by Him, is intended by Him, as a means to enable you to understand wisdom secretly, that you may show forth the fruits of it openly.—*F. D. Maurice.*

We must ask God for patience and faith and surrender every morning; and so the peace of God will keep our hearts and minds, and we shall learn to take all the discipline of life as part of God's education. So shall we find there will grow up in our heart, by degrees, such faith and submission and patience, by the power of God the Holy Ghost, that we shall be delivered from the fretting disquiet which disturbs so many souls in this difficult age.—*Bishop Wilkinson.*

Beyond the rising and the setting
I shall be soon,
Beyond the calming and the fretting,
Beyond remembering and forgetting
I shall be soon.
Love, rest, and home!
Sweet hope;
Lord, tarry not, but come.

*Clewer Manual.*

### Notes and Queries.

**The Editor is always willing to answer in this column, withou any fee, all reasonable questions, as soon as possible.**
**But the following rules must be carefully observed:—**
**1. Every communication must be accompanied by the name and address of the writer.**
**2. The question must always bear upon nursing, directly or indirectly.**
**If an answer is required by letter a fee of half-a-crown must be enclosed with the note containing the inquiry.**

*Baby's Food.*

(119) Will you kindly tell me of something for my little patient's indigestion and acidity? The child is four months old, and I have tried Nestlé's unsweetened milk, Benger's food, Whey, Plasmon, &c. Each food seems to suit the child for a few days, then the sickness returns. I am giving 30m. brandy and a teaspoonful of lime-water every 24 hours.—*K. West.*

We cannot prescribe. Consult a doctor.

*Medical Treatment and Books.*

(120) 1. Can anything be done for sprained muscles under the right arm? They are about as large as marbles, and move about when touched. Would a gathering on the breast have anything to do with the trouble, and what would be the cause of the gathering? 2. What would be the best books for a nurse with one year's training to study, who is about to take entire charge of two tuberculous children?—*Constant Reader.*

1. The presumption is that what "Constant Reader" calls sprained muscles are enlarged glands. How can we pretend to tell "what would be the cause of the gathering." We do not even know whether the patient was man, woman, or child! 2. The question as to books depends on what it is the nurse wants to study. So far as the management of her cases is concerned, she will, of course, have to carry out the directions of the doctor.

*Padding Splints.*

(121) Would you kindly tell me if splints with holes—such as Clines—should be padded over the holes, or, if the holes should be left uncovered?—*Forgetful.*

If they are merely padded with cotton wadding or other such unwoven fabric as can stretch freely, the holes may be covered. But if the padding is enclosed in a woven fabric, this should either be omitted over the holes or a cross cut should be made in it opposite them, extending to the edges of the holes at each side, so as to take off all pressure.

*Book.*

(122) Would you kindly recommend a reliable book on skin diseases, and tell me the price of it.—*E.M.*

We do not know of any special book on the nursing of skin diseases. On the medical side of the question, the section on skin diseases in Allbutt's "System of Medicine" is very good, as also is Malcolm Morris's "Diseases of the Skin" (Cassell and Co. Price 10s. 6d.)

*Post-Graduate Study.*

(123) Would you kindly let me know which are the best London fever hospitals for a qualified medical man to attend externally for a month. And also the fees for that length of time?—*Bertha.*

The hospitals of the Metropolitan Asylums Board. Apply to the clerk.

*Finsen Light.*

(124) Would you kindly tell me if there is any other institution in London besides the London Hospital which possesses the apparatus for the Finsen light cure of lupus. If not would a patient able to pay have to wait long before being permitted to attend the "London" as an out-patient?

*Nurse O.*

Finsen's method of treatment has now been widely popularised, and is in use at several places besides the London Hospital, and if able to pay your medical man would probably be able to make the necessary arrangements. At the London we believe they are "full" for many months to come.

*Baby's Bowels.*

(125) Should anything be done for a breast-fed baby who has five, six, or seven motions in the twenty-four hours? They are normal in colour, thin and frothy, yet the child gains in weight and is apparently healthy. The mother suffers from constipation. 2. May lime-water be given without milk to a breast-fed baby suffering from acidity and diarrhœa?—*Nurse C.*

Not of necessity. If the child ails consult a doctor. Clearly it is not quite right. 2. Yes, a little may be given. But if all is right a child ought not to require it.

*Smallpox Infection.*

(126) It is stated in THE HOSPITAL of April 13th that three cases of smallpox have been traced to infection in Glasgow. Could you inform me in what way it was traced, or where I could obtain the information? We have a young man in this hospital and are trying to trace the source whence he became infected. He has been receiving letters from Glasgow.—*S. T.*

It is very difficult for the residents at a hospital to trace an infection which must have taken place long before the patient's admission. Such inquiries are mostly undertaken by the sanitary authorities, and it must be confessed that they often find the task difficult enough.

**Standard Books of Reference.**

**"The Nursing Profession: How and Where to Train." 2s. net; post free 2s. 4d.**
**"Burdett's Official Nursing Directory." 3s. net.; post free, 3s. 4d.**
**"Burdett's Hospitals and Charities." 5s.**
**"The Nurses' Dictionary of Medical Terms." 2s.**
**"Burdett's Series of Nursing Text-Books." 1s. each.**
**"A Handbook for Nurses." (Illustrated.) 5s.**
**"Nursing: Its Theory and Practice." New Edition. 3s. 6d.**
**"Helps in Sickness and to Health." Fifteenth Thousand. 5s.**
**"The Physiological Feeding of Infants." 1s.**
**"The Physiological Nursery Chart." 1s.; post free, 1s. 3d.**
**"Hospital Expenditure: The Commissariat." 2s. 6d.**
**All these are published by THE SCIENTIFIC PRESS, Ltd., and may be obtained through any bookseller or direct from the publishers, 28 and 29 Southampton Street, London, W.C.**

